# Art-Based Intervention to Promote Psychological Adjustment, Self-Concept, and Quality of Life in Children: Protocol for a Cluster Nonrandomized Controlled Trial

**DOI:** 10.2196/84994

**Published:** 2026-08-12

**Authors:** Sandra Henriques, Ricardo Pinto, Jorge Oliveira, Filipa Sampaio

**Affiliations:** 1 Associação para o Apoio à Integração Social e Comunitária - Espaço T Porto Portugal; 2 HEI-Lab- Human-environment Interaction Lab Faculty of Psychology and Education University Lusófona, University Center of Porto Porto Portugal; 3 Department of Public Health and Caring Sciences Uppsala University Uppsala Sweden; 4 Informatics, Management and Ethics Karolinska Institute Solna Sweden

**Keywords:** cost-effectiveness, emotional adjustment, quality of life, child health, community galleries, self-concept

## Abstract

**Background:**

Children’s mental health is a critical public health priority, with approximately 8% of children aged 10 years or younger experiencing mental, behavioral, or emotional disorders. Despite evidence supporting early intervention, access to mental health services remains limited, particularly for socioeconomically disadvantaged populations. Art-based interventions have emerged as promising approaches to support mental health and socioemotional development. However, significant gaps persist in understanding their efficacy, scalability, and long-term impact.

**Objective:**

This study aims to evaluate the effectiveness and cost-effectiveness of a community-based visual arts intervention on children’s self-concept, psychological adjustment, and health-related quality of life (HRQoL) in vulnerable communities.

**Methods:**

This study is a cluster nonrandomized controlled trial with children from socioeconomically disadvantaged neighborhoods. Children aged 6 to 12 years (n=156; intervention: n=104, 66.7%; waitlist control: n=52, 33.3%) will be recruited through schools and community projects. Allocation to the intervention or waitlist control group will be based on neighborhood or cluster membership. The intervention consists of 1-hour weekly visual arts sessions over 9 months led by a professional artist, culminating in community art exhibitions. Assessments will be conducted at baseline (T1) and postintervention assessment (T2) using validated measures: the Strengths and Difficulties Questionnaire (SDQ) for psychological adjustment, the Self-Perception Profile for Children (SPPC) for self-concept, and the Child Health Utility 9D (CHU9D) for HRQoL, as well as societal resource use and related costs. Quantitative data will be analyzed using regression models and intention-to-treat principles.

**Results:**

This study was funded in September 2023. Recruitment began in March 2024 and ended in July 2025. As of April 2024, a total of 179 participants had been enrolled. Data collection was completed in July 2025, and analysis is expected to begin in April 2026. Results are anticipated to be published in September 2026.

**Conclusions:**

This project has the potential to enhance children’s self-concept, psychological adjustment, and HRQoL through participation in a community-based visual arts intervention. The study design, with assessments at preintervention and postintervention time points, will provide valuable insights into the trajectories of psychosocial and quality-of-life outcomes in this population. In addition, the combination of psychosocial assessment and economic evaluation will generate important evidence on the cost-effectiveness of art-based interventions, informing both educational and public health decision-making.

**Trial Registration:**

ClinicalTrials.gov NCT07165704; https://clinicaltrials.gov/study/NCT07165704

**International Registered Report Identifier (IRRID):**

DERR1-10.2196/84994

## Introduction

### Background

Children’s mental health has been recognized as a critical public health priority, with the latest reports showing that approximately 8% of children younger than 10 years experience mental, behavioral, or emotional disorders [1]. Most children never seek nor receive adequate treatment at a sufficiently early age [2], causing a significant negative impact on familial, peer, and academic functioning, worsening comorbid conditions, and lowering quality of life [3,4]. Mental health problems are common and frequently emerge early in childhood with the potential for lifelong impact [5,6]. Childhood is a sensitive period because the developing brain is more susceptible, making mental health care and support most successful when received early [1]. Thus, even in high-income countries, children often face challenges in accessing mental health services, which tend to be inconsistently available and vary in quality [4].

To address these issues, several conventional methods have been used to promote children’s mental health and prevent mental illness, such as school and community-based interventions [7,8], digital health–based interventions [9], and physical activity interventions [10]. Additionally, one promising approach is the use of the arts as a tool [11,12]. A growing body of research has explored the impact of art-based interventions on both physical and mental health, well-being, and quality of life across the lifespan. Research indicates that participating in art activities can promote various outcomes, such as heightened social interaction, improved emotional regulation, reduced mental distress, and better socioemotional growth [11,13-17]. One possible explanation is that art-based interventions enhance a person’s ability for creative thought and action [18], facilitate nonverbal communication, promote self-expression, and foster social connectedness, which may contribute to alleviating emotional problems [19], enhancing self-esteem [20] and self-concept [21], and improving psychological adjustment [22-24].

Despite the promising findings, significant gaps remain in understanding the efficacy of art-based mental health interventions. A primary limitation is the small sample sizes commonly used in studies, which restrict the generalizability of findings and hinder the ability to establish causality [25]. Additionally, there is limited evidence regarding the scalability and long-term efficacy of such interventions, which constrains their broader implementation and impact [11]. Furthermore, little is known about the specific impact of specific visual arts interventions on socioeconomically disadvantaged populations, particularly children [26]. These disadvantaged groups seem to face particular challenges, such as low income, high prevalence of parent’s mental health problems, low education, and are more likely to experience adversity and related life events (RLE) [1].

### Study Setting

The present study is set in Porto, the second-largest city in Portugal, and targets children living in socioeconomically disadvantaged neighborhoods. According to Statistics Portugal, a significant proportion of children live below the poverty threshold, with higher concentrations in metropolitan areas such as Porto [27]. Portugal’s mental health care system has an insufficient availability of community services, particularly affecting children and youth [28,29]. Epidemiological studies on the diagnosis, prevalence, and access to mental health services for children and adolescents are scarce in Portugal, and there is a recognized need to integrate schools and community-based programs into mental health care to improve access [29-31].

Addressing these gaps is essential to advance the understanding of how art-based interventions can support mental health, particularly in vulnerable populations. Therefore, this study seeks to address these gaps by using a longitudinal approach to investigate the impact of art-based interventions on children from vulnerable populations, focusing on their socioemotional development and psychological adjustment.

### Study Aims

This study aims to evaluate the potential benefits of an art-based intervention in improving children’s self-concept, psychological adjustment, and health-related quality of life (HRQoL). Specifically, the primary objective is to assess the impact of the intervention on children’s psychological adjustment, and the secondary objectives are to assess the effect of the intervention on children’s self-concept and HRQoL and to investigate the cost-effectiveness of the intervention compared with a waitlist control. By addressing these aims, the study will provide evidence on the effectiveness and cost-effectiveness of an art-based intervention as a tool for promoting mental health and social integration among children in disadvantaged settings.

## Methods

### Design

The study design is a cluster nonrandomized controlled study with 2 arms: the intervention group, which includes neighborhoods participating in different activities, and a waitlist control group, which includes neighborhoods starting the intervention in year 2. The nonrandomized design was adopted because of ethical and structural constraints inherent to the community-based setting. Clusters were preexisting and defined by schools and community projects located in distinct neighborhoods. Because the participating institutions were already organized as groups, students or participants could not be redistributed or reorganized for randomization purposes. Individual randomization was also not feasible, as the intervention was delivered in a group format within each institution, that is, to a class or group simultaneously. Data collection ran from March 2024 to July 2025, and data cleaning and analysis started in April 2026.

### Participants

The target population will be children aged 6 to 12 years. To be eligible for the study, children must be enrolled in primary school and attending one of the 10 project institutions or partner schools and residing in socially deprived neighborhoods in Porto identified as being at risk of social exclusion. Children with a cognitive deficit, a neurological disorder, or a global developmental disorder will not be eligible.

On the basis of an expected medium effect size (Cohen *d*=0.5), an α error probability of .05, and 90% statistical power, using the difference of means between 2 independent samples with a 2:1 allocation ratio, we estimate that a total of 156 participants will be required to detect significant results (n=104, 66.7% in the experimental group and n=52, 33.3% in the control group). We anticipate fewer participants in the control group at the end of the study due to its higher likelihood of dropout [32]. Participants in the control group are not directly involved in the intervention, which may decrease their perceived benefit, reduce their engagement, and increase the possibility of withdrawal compared with those actively participating in the experimental intervention. However, if this occurs, the results will not necessarily be biased [33]. This consideration justifies the use of a 2:1 allocation ratio, ensuring sufficient statistical power despite the potential for unequal group retention rates. To account for an estimated 30% dropout or refusal rate, we will initially recruit approximately 223 participants.

### Procedures

The researchers will coordinate with established partner schools and social projects in 10 neighborhoods to disseminate information about the study’s objectives and procedures. Local partners will inform caregivers about the study’s purpose and procedures, invite them to participate, and obtain informed consent from legal guardians for those willing to participate.

Furthermore, verbal assent will be sought from the children themselves to ensure their willingness to participate. Data collection will preferably be conducted in person. Online data collection will only be carried out if the participant cannot complete the form in person or because of a justified absence and requests online completion. In cases where online completion is requested, the participant’s or guardian’s email will be collected, and a link will be sent to access the Qualtrics platform. Identifiable data, such as email addresses, will never be stored together with the raw data. To minimize any differences between the modalities, the collection method will be recorded for later analysis. Statistical control of any effects of the modality on the results will also be considered. Only those children whose parents or legal guardians have provided informed consent and who have also given their own verbal assent will be included in the study. Due to the noninvasive and low-risk nature of the intervention, a formal data monitoring committee is not required. Any minor issues arising during the sessions will be addressed immediately through internal safety procedures. To ensure data confidentiality, the following procedures will be implemented: (1) using a system code for each research protocol, (2) separating the research protocol and informed consent to maintain participants’ anonymity, (3) collecting only personal data necessary for research purposes, and (4) entering data in statistical software and analyzing them solely in a collective manner. We will apply Portuguese law and European Union directives regarding personal data protection. Data will be anonymized and stored on encrypted university-based electronic servers, with restricted access to the research team. Participants’ personal information will not be shared with third parties. Deidentified datasets will be made accessible in a repository for verification and reuse. All procedures, objectives, and expected contributions will be communicated in advance to the participants and their guardians. However, interested parties will be able to obtain more detailed information about the study by contacting the principal researcher by email. Additionally, after the study’s conclusion, an information meeting will be held at the partner institutions, to which guardians will be invited. The results and conclusions of the study will be made available to participants and their guardians through the institutions or schools involved, where they will be able to access a QR code linking to a webpage containing the reports, articles, and communications of the study. Flyers with a summary of the main results will be delivered to the partner institutions for distribution to the children and legal guardians.

**Figure 1:**
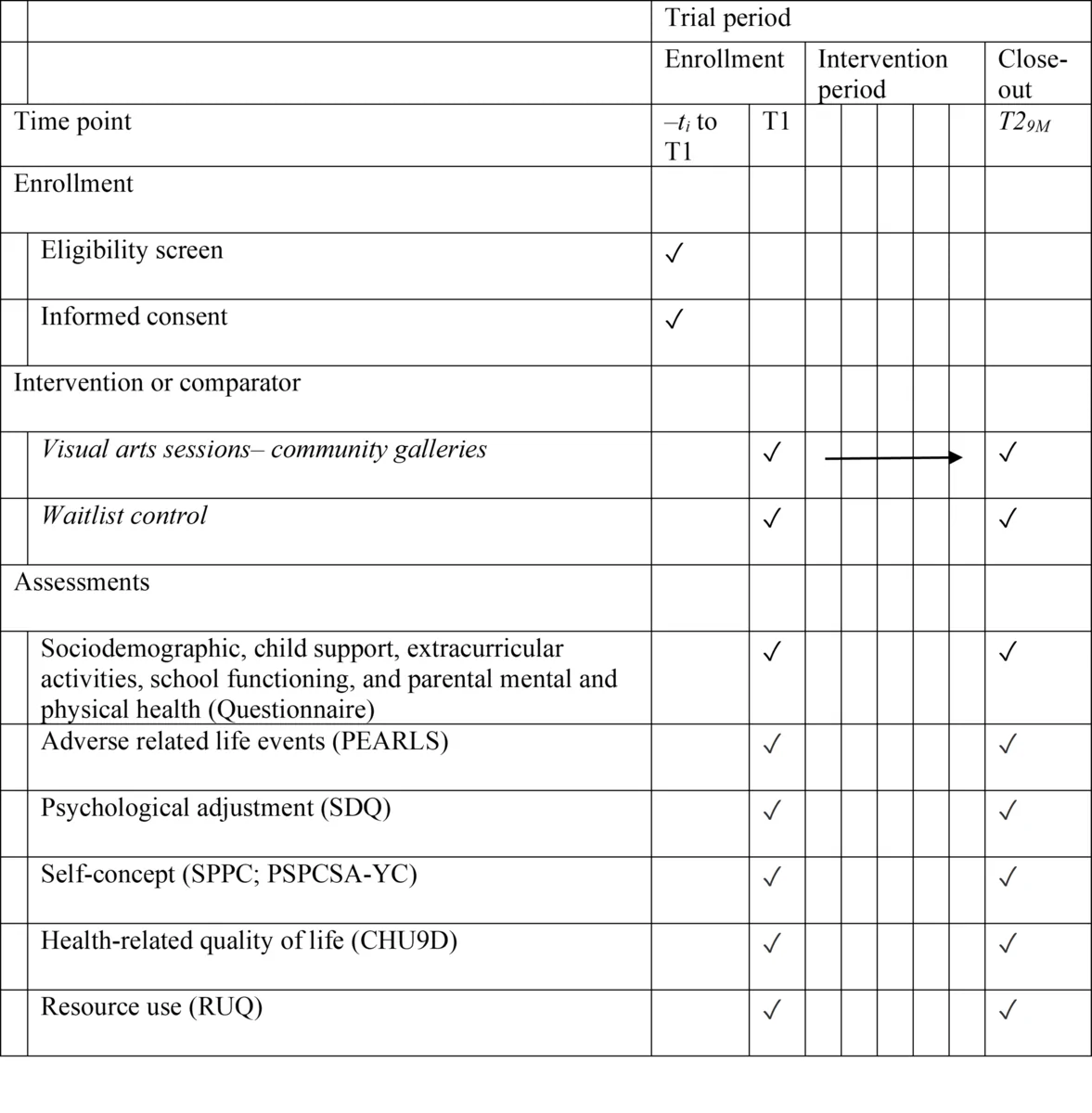
Participant timeline—schedule of enrollment, interventions, and assessments. CHU9D: Child Health Utility 9D; PEARLS: Pediatric Adverse Childhood Experiences and Related Life Events Screener; PSPCSA-YC: Pictorial Scale of Perceived Competence and Social Acceptance for Young Children; RUQ: resource use questionnaire; SDQ: Strengths and Difficulties Questionnaire; SPPC: Self-Perception Profile for Children.

This trial includes 2 assessment waves: (T1) baseline and (T2) postintervention assessment, 9 months after baseline. A participant timeline is provided in Figure 1. At baseline (T1), caregivers and children will provide sociodemographic information, details on family mental health history and relevant adverse RLE, and information on resources used by participants. Given that some children may experience difficulties with reading and interpreting questions, all children will rate their self-concept and HRQoL, ensuring uniformity in data collection across participants. Additionally, teachers or social project technicians (depending on the cluster) will report on the children’s behaviors, emotions, and relationships. Following the baseline assessments, participants will be assigned to either the control or intervention group without randomization. The nonrandomized assignment arises because the clusters, defined by schools or social projects, are fixed and cannot be restructured for randomization purposes. At postintervention assessment (T2), the same protocol as at baseline (T1) will be followed: caregivers will provide details on the family mental health history, relevant adverse RLE, and resources used by participants. Children will also rate their self-concept and HRQoL, while teachers or social project technicians will report on the children’s behaviors, emotions, and relationships.

### The Art Intervention Community Galleries

The art intervention will consist of 2 parts. First, implementation of a 1-hour visual arts session (eg, painting, drawing, and printing techniques exercises) once a week for 9 months with the experimental group. Sessions are delivered in person, in real time, in a group format of 10 to 20 children per venue, across 10 community venues situated in socially deprived neighborhoods of Porto. The activities will be guided by a theme (emotions) by a qualified art facilitator (artist) with prior experience working with children to obtain a final individual artistic product. The session structure is adaptive: while the thematic focus and the artistic medium remain consistent, the facilitator adjusts pacing, complexity, and sequencing in response to participants’ receptivity and motor and cognitive capacities, given that children range in age. Each session typically includes (1) an introductory phase in which the facilitator introduces the theme and demonstrates a technique using materials such as paints, watercolors, pencils, canvas, paper, and accessible printmaking plates; (2) an active creation phase in which participants produce individual artwork independently within the group setting; and (3) an informal sharing phase in which children exchange ideas, difficulties, and improvements with peers. Through strategies such as repetition, observation, and imagination, the exercise promotes a spirit of inquiry and learning. Peer contact, shared learning, and a feeling of community are all made possible by the group-based approach. The facilitator will provide support, motivation, and technical training while guiding participants through the creative process and will also monitor adherence by collecting signatures to confirm attendance at the sessions. Using narrative-building and dialogue-based methods, the artist will encourage children to share their stories, experiences, and perspectives through art. Narrative-based techniques use narrative to investigate human experiences, emotions, and perspectives. By constructing tales via art, individuals may make sense of their experiences, manage complicated emotions, and get a better understanding of themselves. Dialogue-based strategies enable people to have meaningful conversations about their experiences. The intervention is guided by several interrelated principles. Self-expression is a foundational orientation, with participants afforded autonomy to represent their emotions and experiences according to their own visual preferences within a technically guided framework. Individual self-worth is promoted through the public display of artwork in community exhibitions. Emotional exploration constitutes the thematic spine of the program, providing a coherent and recurring psychological framework across all sessions. The session design also promotes present-moment engagement through attentive sensory involvement with materials, including awareness of hand movements, textures, and visual composition, as well as aesthetic appreciation of color, form, and harmony. Creativity and personal narrative are central to the activity design, and the group format fosters a sense of belonging and shared identity. The intervention is expected to promote emotional regulation through sustained engagement with emotion-focused creative tasks, self-concept through the experience of competence and the valorization of each child’s artistic production, relational security through the consistency of the same facilitator throughout the project and the stable composition of preexisting peer groups, and group cohesion and agency through collaborative sharing, peer learning, and guided autonomous creative choice-making.

The program was conceived and developed by a multidisciplinary team comprising a cultural producer, a qualified art facilitator with 2 years of prior field experience working with children in the same community neighborhoods, and a psychologist.

Second, children’s final artistic products will be displayed in the community galleries in their neighborhood in an opening ceremony. All primary school students in Portugal follow a national curriculum that includes arts education as a mandatory subject, known as “Expressive Arts,” meaning that both the intervention and waitlist control group will have some exposure to arts activities throughout the study period. However, while the regular Expressive Arts curriculum is delivered by class teachers as part of the curriculum, our program is a structured, community-based enrichment initiative designed by a professional artist, with explicit socioemotional development goals, using materials and arts methods that differ from what is available at schools and the standard curriculum.

### Measures

The study’s design, assessment waves, variables, and measures are presented in Table 1.

**Table 1:** Study design, assessments, variables, and measures.

	Baseline (T1)	Postintervention assessment (T2)
Parent or caregiver	Sociodemographic, child support services, extracurricular activities, school functioning, and parental mental and physical health historya Children’s adverse related life events exposure (PEARLSb) Resource use (RUQc)	Sociodemographic, child support services, extracurricular activities, school functioning, and parental mental and physical health history a Children’s adverse related life events exposure (PEARLSb) Resource use (RUQc)
Teacher or technician	Children’s psychosocial adjustment (SDQd)	Children’s psychosocial adjustment (SDQd)
Child	Self-concept (PSPCSA-YCe and SPPCf) Health-related quality of life (CHU-9Dg)	Self-concept (PSPCSA-YCe and SPPCf) Health-related quality of life (CHU-9Dg)

^a^Questionnaire on sociodemographic characteristics, child support services, extracurricular activities, school functioning, and parental mental and physical health history.

^b^PEARLS: Pediatric Adverse Childhood Experiences and Related Life Events Screener.

^c^RUQ: resource use questionnaire.

^d^SDQ: Strengths and Difficulties Questionnaire.

^e^PSPCSA-YC: Pictorial Scale of Perceived Competence and Social Acceptance for Young Children.

^f^SPPC: Self-Perception Profile for Children.

^g^CHU-9D: Children’s Health Utility-9D.

#### Sociodemographic Data

The caregiver will report on themselves and on the child’s age and gender, as well as their own education, marital status, employment status, perceived relative income, housing status, nativity or residence, and number of biological and nonbiological children.

#### Self-Reported Mental Health History

Caregiver’s mental health will be assessed through self-report items on the history of mental health diagnosis, current mental health problems, and receipt of treatment. Response options will include self, other parent or caregiver, both, no, do not know, and prefer not to answer. For those receiving treatment, the type of treatment was recorded (medication, professional support or psychotherapy, both, or other).

#### Child Support Services, Extracurricular Activities, and School Functioning

The caregiver will report on the child’s receipt of support services (current or past), specifying the types of support received. Additionally, caregivers indicate the child’s current participation in extracurricular activities, including the frequency per week. School-related information will also be collected, encompassing grade repetition, school attendance (including the number of unexcused absences), and parent-rated assessments of the child’s behavior and academic performance.

#### Adverse Related Life Events

Parents will report on their children’s RLE, which will be assessed using part 2 of the Pediatric Adverse Childhood Experiences and Related Life Events Screener (PEARLS) [34]. The 7 questions include physical illness or disability of a caregiver, death of a caregiver, housing instability, forced separation from caregiver, exposure to discrimination, food insecurity, and community violence. We will categorize the 7 RLE responses as “no RLEs,” “1-3 RLEs,” and “≥4 RLEs.”

### Primary Outcome

The Strengths and Difficulties Questionnaire (SDQ [35,36]; Portuguese version [37]) is a behavioral screening tool comprising 25 items and an impact supplement. Intended for educators and teachers, it is applicable to children, with a specific version available for school-age children (aged 4 to 17 years) [36]. On the basis of the child’s behavior in the last 6 months, respondents are required to mark 1 of 3 responses: “not true,” “somewhat true,” and “certainly true.” The 25 items are distributed across 5 scales, with 4 representing problematic behaviors (emotional symptoms, conduct problems, hyperactivity, and problems with peer relationships), and one scale assessing prosocial behavior. Results are obtained by summing the values of the items in each scale and are interpreted as normal, borderline, or abnormal based on standardized values (minimum 0 and maximum 10 for each scale). The impact supplement, intended for educators and teachers, addresses the chronicity of the problem, level of stress, social adjustment of the child, and burden associated with the problem for others. This questionnaire has been widely used in research on externalizing behavior problems. Studies indicate internal consistency ranging between 0.65 and 0.85 for the teachers’ version [38].

### Secondary Outcomes

#### Self-Concept

The Self-Perception Profile for Children (SPPC [39]; Portuguese version [40]) is a 36-item self-report instrument designed to assess the perceptions of children aged 8 to 13 years regarding their own competence across 6 domains. Developed by Susan Harter in 1985 [39], the Portuguese version is an adaptation for the Portuguese-speaking population. The SPPC covers multiple domains of self-perception, including scholastic competence, social acceptance, athletic competence, physical appearance, and global self-worth. It allows children to rate themselves on a scale, providing insights into their perceived competencies in each area. Each item is presented in a structured format, asking the child to choose which of 2 opposing statements is more like them (eg, “Some kids feel they are very good at their schoolwork, BUT other kids worry about whether they can do the work assigned to them”), followed by a choice of “really true for me” or “sort of true for me.”. In the original version, Harter [39] reports internal consistency (Cronbach α) values ranging from 0.71 to 0.86 across subscales, a range further corroborated in cross-cultural replications [41]. The Portuguese adaptation by Faria (2001) [42] reported Cronbach α coefficients between 0.25 and 0.80, reflecting acceptable to good reliability in Portuguese-speaking samples. The 6-factor structure has shown good construct validity across multiple samples and cultures, correlating meaningfully with external measures of psychopathology and personality [41]. In the present study, the SPPC will be administered to children aged 8 to 12 years (ie, from the third grade onward), while the Pictorial Scale of Perceived Competence and Social Acceptance for Young Children (PSPCSA-YC) [43] will be used for younger children aged 6 to 7 years, in line with the age-differentiated recommendations of the 2 instruments.

The PSPCSA-YC (Portuguese version [44]) is a 24-item validated instrument to measure self-perceptions among children aged 4 to 7 years. This scale assesses perceived competence and social acceptance among children in 4 areas: cognitive competence, physical competence, peer acceptance, and maternal acceptance. For every item, the children are shown 2 pictures that depict opposite situations (for instance, one picture shows a child who can do a task, and the other picture shows a child who cannot do a task), together with a short description. The child chooses which picture they identify with and then indicates whether the statement is “totally true” or “quite true” for them, resulting in a 4-point scale for each item. In the original validation study, Harter and Pike [43] reported internal consistency coefficients (Cronbach α) ranging from 0.62 to 0.83 across the 4 subscales. The Portuguese adaptation reported a Cronbach α of 0.87 for the total scale and values ranging from 0.68 to 0.82 across subscales [45], while the Portuguese version used in the present study [44] further confirmed the 4-factor structure for Portuguese-speaking preschool and early school-age populations.

Health-Related Quality of Life The Child Health Utility 9D (CHU9D [46]; Portuguese version) is an HRQoL measure designed for use in children, reflecting the multifaceted nature of health in children and youth aged 7 to 17 years [47]. However, it can be used in children as young as 6 years, with interviewer assistance [48]. It has demonstrated acceptable to good psychometric properties across pediatric populations, including internal consistency, with Cronbach α ranging from 0.70 to 0.82 [49-51]. Evidence also supports its test-retest reliability, with moderate to strong stability over time [50,52]. Construct validity has been confirmed through the expected factor structure in confirmatory factor analyses and moderate to strong correlations with established pediatric HRQoL instruments, such as the PedsQL and KIDSCREEN (*r*=0.34-0.70) [50,53]. The CHU9D captures 9 dimensions: worry, sadness, pain, tiredness, annoyance, schoolwork, sleep, routine, and activities. For each dimension, a 5-level ordinal scale is used, ranging from 1 (no problems) to 5 (severe problems). Intended for use in cost-effectiveness analyses of youth-focused treatment and assistance initiatives [49], the instrument derives a single utility score of the health state experienced by the child from preference weights. CHU9D utility scores will be used to estimate quality-adjusted life years (QALYs) for children over the study period. In this study, we will use the self-report version.

#### Resource Use

Data on resources used by children and their caregivers will be collected at both time points via a resource use questionnaire (RUQ), adapted from questionnaires by Bouwmans et al [54] and Pokhilenko et al [55]. The RUQ will collect information on health care services (accident and emergency visits, outpatient visits, and inpatient stays), medication, child protection services, absenteeism and presenteeism from school, the use of support lines, absenteeism and presenteeism from both paid and unpaid work, and out-of-pocket expenses for caregivers.

### Data Analysis

Descriptive statistics will be calculated for all variables to summarize sample characteristics and study measures.

#### Matching Methods

Due to the study’s nonrandomized nature, there may be selection bias, whereby differences between groups (eg, age and health status) may influence outcomes, making it more challenging to isolate the effect of the intervention. To reduce potential selection bias, we will explore the use of propensity score methods to improve baseline comparability between the intervention and waitlist control groups. Propensity scores will be estimated using preintervention covariates selected a priori based on their relevance to group allocation and study outcomes. Covariate balance will be assessed using standardized mean differences and inspection of propensity score overlap. If adequate balance is not achieved or if matching substantially reduces the sample, regression-based analyses controlling for the same covariates will be used. Results will be interpreted in light of any remaining imbalance and potential residual bias [56].

#### Mixed-Effects Model Analysis

Data analysis will follow the intention-to-treat and per-protocol principles and the Standard Protocol Items: Recommendations for Interventional Trials (SPIRIT) statement guidelines for reporting and analyzing trials [57]. The confirmatory analysis of the primary end points (psychological adjustment, self-concept, and quality of life) will use mixed-effect regression modeling (hierarchical linear multilevel models) that accounts for the longitudinal structure and adjusts for potential confounders. The response variables will be the total scores and subscales of measures assessing conduct problems and self-concept, while the interaction terms between time and groups will represent the adjusted effects of the visual arts intervention and will be presented as the main study findings (hierarchical comparisons of treatment conditions with the control group). The models will also include a random intercept for the cluster to account for the nested structure of the data.

#### Health Economic Evaluation

The health economic evaluation will follow the Consolidated Health Economic Evaluation Reporting Standards 2022 Statement [58]. A cost-utility analysis will be conducted using QALYs for children as the outcome (measured using CHU9D scores). CHU9D utility scores will be used to estimate QALYs for children over the study period using the area under the curve method [59]. Costs will be calculated from both the perspective of the Portuguese health care payer (Serviço Nacional de Saúde) and a broader societal perspective. The health care perspective will include health care costs accruing to the payer, whereas the broader perspective will include additional costs beyond the health care sector. The difference in QALYs between the intervention and control groups will be compared with the difference in costs and presented as an incremental cost-effectiveness ratio, expressed as the cost per QALY gained. Uncertainty around the cost and QALY estimates will be presented on a cost-effectiveness plane. Net monetary benefits at different willingness-to-pay thresholds will also be calculated. A cost-effectiveness acceptability curve will be used to illustrate decision uncertainty and depict the likelihood of the intervention being cost-effective across various thresholds [60].

#### Missing Data

Case and item missing data will be evaluated to assess the nature of the missingness and to subsequently determine the most appropriate methodology. Multiple imputation by chained equations with predictive mean matching may be used to reduce bias from missing responses in the main intention-to-treat analysis if the data are missing at random [61]. The required number of imputed datasets will be based on the percentage of missing data and determined by implementing the “how_many_imputation” command by von Hippel [62]. As the economic data are likely to be missing at random, multiple imputation will be used in the base-case analysis [62].

### Ethical Considerations

All aspects of this research project comply with the ethical standards of the Ethics Committee of the Faculty of Psychology and Education of Lusófona University, Porto (CEDIC122_04.25). All participants were given clear information about the purpose of the study, what their participation would involve, and their rights. Informed consent was obtained from parents and legal guardians prior to data collection. Verbal assent was obtained from all participants. Privacy and confidentiality were carefully protected throughout the study. Any personal information was anonymized, and all data were stored securely to ensure that it could not be accessed by unauthorized individuals. No participant compensation was provided. Any major alteration to the procedures will be communicated to the sponsor and submitted to the Ethics Committee. Study findings will be shared with both the scientific and nonscientific communities. Periodic newsletters, such as fact sheets, research briefs, and results, will be created and shared on relevant social networks by the nonscientific community. Results will be presented to and shared with the scientific community through international articles and national and international research activities.

## Results

**Figure 2:**
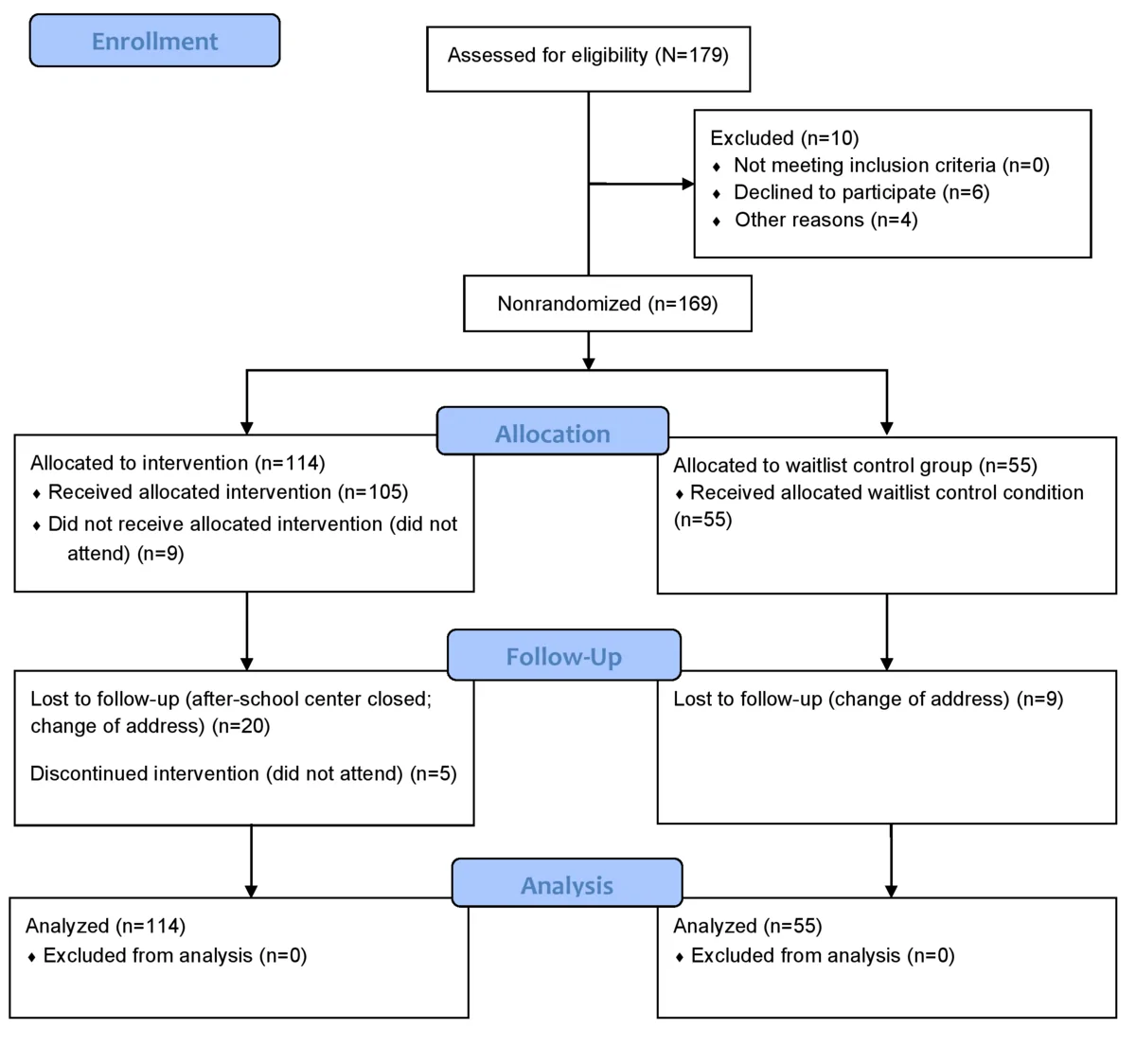
CONSORT (Consolidated Standards of Reporting Trials) flow diagram.

The project was funded in September 2023. Participant recruitment started in March 2024 and concluded in July 2025. As of April 2024, a total of 179 participants had been enrolled. Data collection was completed in July 2025, and analysis is expected to begin in April 2026. The final results are anticipated to be published in September 2026. A CONSORT (Consolidated Standards of Reporting Trials) flow diagram is presented in Figure 2.

## Discussion

### Anticipated Findings

This protocol describes a cluster nonrandomized controlled trial to assess a community-based visual arts intervention for children. We hypothesize that participating in a visual arts intervention will be associated with improvements in psychological adjustment, self-concept, and HRQoL compared with a waitlist control group. A cost-effectiveness analysis will also be conducted to estimate the cost per QALY gained. By encompassing the psychosocial and economic domains together, this study can make an important contribution to the evidence base on art-based intervention for children’s psychological adjustment and self-concept. The findings could inform scalable, low-cost, and culturally adaptable strategies for promoting better outcomes for children in disadvantaged settings. If successful, the Community Galleries—Art and Children in Action approach could be extended on a broader scale as part of educational and public health policies.

Previous studies have been characterized by small sample sizes, short intervention periods, and methodological designs that limit generalization and causal inference [11,25]. Furthermore, little is known about the specific impact of community-based visual arts interventions on children’s mental health, specifically in socioeconomically vulnerable contexts [26]. In addition, economic evaluations remain scarce in this field [50]. Our study will address these gaps by using a waitlist control group for comparison, a larger sample, an intervention period of 9 months, validated and standardized outcome measures, and the inclusion of a cost-utility analysis. We also aim to prioritize ecological validity by implementing the intervention in natural school contexts, which will enhance the transferability of findings to real-world contexts.

### Strengths and Limitations

The use of standardized and validated assessments (SDQ, SPPC and PSPCSA-YC, and CHU9D) will ensure robust measurement of key psychosocial and quality-of-life outcomes, with potential for comparability across other research in this area. In addition, the inclusion of a cost-utility analysis, based on QALYs derived directly from CHU9D scores, addresses an essential gap in the literature on the economic value of arts interventions [63] and will provide decision-makers with key evidence to support resource allocation in this area.

Despite these strengths, some methodological limitations have to be considered. The nonrandomized study design, limited by the fixed nature of the involved schools and neighborhood projects, could restrict the ability to make causal inferences. Similar methodological limitations have been highlighted in previous art-based intervention studies, including restricted causal inference and limited generalizability [11,25]. Although the nonrandomized design limits causal inference, the intervention is implemented in naturalistic school and community settings, which enhances ecological validity and increases the relevance of the findings to similar real-world contexts, while also supporting the assessment of scalability. Cluster allocation may further reduce between-group contamination by ensuring that participants within the same setting are assigned to the same condition. To strengthen the validity of group comparisons, all participants will be analyzed according to their original allocation, regardless of intervention completion, in line with intention-to-treat principles, thereby reducing bias associated with selective dropout. In addition, we will explore the use of matching methods to improve comparability between groups and reduce baseline imbalances inherent in the nonrandomized design. The risk of contamination between intervention and control clusters is considered limited, given the geographic separation of the neighborhoods in which the participating schools are located. However, we acknowledge that variability in how individual teachers implement the standard *Expressive Arts* curriculum across schools may introduce some heterogeneity between groups; this has been noted as a potential limitation of the study. A further limitation is the absence of follow-up beyond the postintervention assessment time point, which limits the ability to assess the durability and long-term sustainability of any observed effects.

### Future Directions

Future studies should incorporate longer follow-up periods to assess the durability of intervention effects and further explore the intervention’s mechanisms of change, such as the role of creative self-expression and emotional regulation as potential mediators. Replication in other contexts will strengthen the evidence for the scalability of community-based visual arts interventions.

### Dissemination Plan

The results of the study will be presented at international conferences in the fields of public health, arts, child mental health, and health economics. They will also be disseminated through scientific publications. Participants, families, partner institutions, and schools will have access to a summary of findings through accessible formats, including a dedicated web page and information flyers.

## Abbreviations

CHU9D: Child Health Utility 9D
CONSORT: Consolidated Standards of Reporting Trials
HRQoL: health-related quality of life
PEARLS: Pediatric Adverse Childhood Experiences and Related Life Events Screener
PSPCSA-YC: Pictorial Scale of Perceived Competence and Social Acceptance for Young Children
QALY: quality-adjusted life year
RLE: related life events
RUQ: resource use questionnaire
SDQ: Strengths and Difficulties Questionnaire
SPIRIT: Standard Protocol Items: Recommendations for Interventional Trials
SPPC: Self-Perception Profile for Children

## Data Availability

No datasets were generated or analyzed during the current study. The protocol will be published open access. Due to ethical and privacy considerations, deidentified participant-level data and statistical code will not be made publicly available but may be shared upon reasonable request to the principal investigator, subject to institutional approvals.
